# Biologically-active exosomes in plasma of AML patients inhibit innate immunity and promote leukemia progression

**DOI:** 10.1186/2051-1426-3-S2-P278

**Published:** 2015-11-04

**Authors:** Michael Boyiadzis, Chang-Sook Hong, Theresa L Whiteside

**Affiliations:** 1 University of Pittsburgh, Pittsburgh, PA, USA

## 

AML patients are reported to have impairments of immune cells which contribute to leukemia progression. Tumor-derived exosomes (TEX) have recently emerged as carriers of the molecular and genetic cargo with potent immunosuppressive properties. We showed that plasma of newly-diagnosed AML patients prior to any therapy contained high levels of exosomal proteins relative to those in plasma of normal donors (NC). AML exosomes were enriched in membrane-associated TGF-β1, MICA/MICB and markers of myeloid blasts. We hypothesize that these plasma-derived virus-size (30-100nm) membrane-bound vesicles operating in AML deliver suppressive signals to immune cells and thus may promote leukemia progression.

## Methods

Venous blood (20-50 mL) was obtained from patients newly diagnosed with AML prior to any treatment, from AML patients who achieved CR following initial therapy and from age-matched NC. Exosome fractions were isolated from plasma using mini-size exclusion chromatography (mini-SEC) with Sepharose 2A. Exosome protein levels, numbers and size of exosomes (qNano) and their morphology (TEM) were determined. Exosomes were characterized by Western blots for expression of exosome markers, Tsg101 and CD81, and myeloid cell-surface markers associated with AML, interleukin-3 receptor alpha chain (CD123) and C-type lectin-like molecule-1 (CLL-1), CD44 and CD96. Isolated normal human NK cells were co-incubated with AML exosomes and multiparameter flow cytometry was used to monitor changes in expression levels of NKG2D on NK cells.

## Results

Exosome fractions isolated from AML patients' plasma at diagnosis (n=16) had higher mean protein content than fractions obtained from NC plasma (96 vs 37 µg protein/mL plasma, p=0.03). Exosomes were isolated from paired AML plasma samples obtained at diagnosis and in CR after chemotherapy (n=13). In the exosome fractions isolated at CR, at the time when leukemic blasts were undetectable in the bone marrow by conventional hematopathological methods, protein levels remained elevated (mean value=120 µg protein/mL plasma). AML exosomes isolated at CR carried blast markers, CD123, CLL-1, CD44, CD96 and were enriched in the mature form of TGF-β1. Co-incubation of these exosomes with activated normal human NK cells resulted in down-regulation of NKG2D expression with a concomitant reduction of NK-cell cytotoxicity. Ten/13 AML patients in CR whose plasma exosome levels remained elevated eventually relapsed.

## Conclusion

The exosome fractions in plasma of patients with AML are enriched in blast-derived exosomes carrying leukemia blast markers and biologically-active TGF-β1. The persistently elevated levels of these exosomes in plasma of AML patients in CR contribute to impaired anti-leukemia immune responses and promote leukemia progression.

